# High prevalence of bronchiectasis on chest CT in a selected cohort of children with severe Asthma

**DOI:** 10.1186/s12890-019-0900-0

**Published:** 2019-07-26

**Authors:** David Lo, Amit Maniyar, Sumit Gupta, Erol Gaillard

**Affiliations:** 1 Robert Kilpatrick Clinical Sciences Building. Leicester Royal Infirmary, University Hospitals of Leicester, Leicester, LE2 7LX UK; 2 Royal Manchester Children’s Hospital, Manchester University Foundation Trus, Manchester, UK; 3 Department of Radiology, Glenfield Hospital, University Hospitals of Leicester NHS Trust, Leicester, UK; 4 NIHR Leicester Respiratory Biomedical Research Unit, University of Leicester. Leicester Institute for Lung Health, Leicester, UK

**Keywords:** Tomography, X-ray computed, Child, Radiology, Bronchiectasis

## Abstract

**Background:**

Chest computed tomography (CT) scans have a recognised role in investigating adults with severe asthma to exclude alternative diagnoses, but its role in children is less clear. The objective of this study was to review the CT findings of our local cohort of children with severe asthma and to explore whether clinical or pathobiological parameters predicted CT changes.

**Methods:**

Retrospective observational single centre study including all children attending the Leicester difficult asthma clinic (DAC) who underwent a chest CT from 2006 to 2011. Additionally, we recruited eight age-matched, non-asthmatic controls to compare differences in CT findings between asthmatic and non-asthmatic children. All CT images were independently scored by two radiologists.

The DAC patients were sub-divided into binary groups for each abnormality identified so that comparisons could be made against recorded clinical variables including age, lung function, serum total IgE levels, and sputum leukocyte differential cell counts.

**Results:**

Thirty DAC patients (median 12 yrs., range 5–16) were included.

The most common abnormalities were bronchial wall thickening (BWT) and air trapping (AT), observed in 80 and 60% of DAC patients.

Bronchiectasis (BE) was identified in 27% of DAC patients. DAC patients with evidence of BE on CT images were older than those without BE (13.9 ± 0.67 vs 11.5 ± 0.61, *p* = 0.038). We also identified a positive correlation between increasing BE severity and extent with age (r = 0.400, *p* = 0.028).

**Conclusion:**

Abnormal CT findings were highly prevalent in our cohort of children with severe asthma, with bronchiectasis identified in approximately one third of children. We found no alternative diagnoses that resulted in a change in clinical management.

## Background

Asthma is the commonest chronic disease of childhood affecting 1.1 million children in the UK [1]. It is estimated that approximately 10% of children with asthma have severe asthma and require high-dose inhaled or oral corticosteroids to control their symptoms [2]. Although this represents a relatively small proportion of the total asthma population, people with severe asthma experience increased morbidity, and account for a disproportionately higher amount of healthcare spending [3, 4].

Both the European Respiratory Society (ERS) and American Thoracic Society (ATS) guidelines recommend high resolution chest computed tomography (HRCT) scanning for adults and children with an atypical presentation of severe asthma, or if there are other specific indications based on the patient’s clinical history, symptoms, or results of other investigations [2]. CT scans, in the context of asthma, can be used to exclude conditions such as bronchopulmonary aspergillosis or parenchymal abnormalities [5], and to detect conditions which mimic asthma such as hypersensitivity pneumonitis [6].

In adult practice, chest CT scans already have a recognised role as part of the diagnostic workup of patients with severe asthma [2, 7]. Studies have demonstrated abnormal radiological findings including bronchial wall thickening (BWT), bronchial wall dilatation, bronchiectasis (BE), hyperinflation, mucus plugging and emphysema [8, 9]. In adults with severe asthma, BWT in particular has been shown to correlate with reticular basement membrane thickening [10]; which is a characteristic feature of airway remodelling and inversely correlates with respiratory function.

However, in children only a small number of studies have reported chest CT findings in patients with severe asthma, and their relationship to clinical and pathobiological parameters. BWT and air trapping (AT) are more common in children with severe asthma [11–13], but the presence of either of these abnormalities have not been shown to be associated with poorer lung function. In terms of BE, a previous study investigating the causes of BE in children identified asthma as a potential causative factor [14], but this study was not specifically looking at the severe asthma cohort.

There is currently limited evidence to support the use of chest CT imaging in children with severe asthma. Unlike in adult studies, previous studies in children with severe asthma have failed to detect an association between chest CT findings and lung function.

Within our paediatric severe asthma cohort, we noted a diagnosis of BE on a number of CT reports. This has not been previously reported in children with severe asthma. The aim of this retrospective observational study was to evaluate whether this was a true finding, and whether abnormal CT changes were associated with measured clinical parameters.

## Materials and methods

### Study design

This was a retrospective single centre observational study conducted between 2014 and 2016.

### Subjects

All children attending the University Hospitals of Leicester difficult asthma clinic (DAC) who underwent a chest CT as part of their clinical evaluation between the years 2006 to 2011 were identified. Children attending the DAC are extensively investigated. This includes an assessment of their clinical history and asthma control, and measurements of spirometry, exhaled nitric oxide levels, serum IgE, serum eosinophil count, and sputum induction for leukocyte differential cell counts prior to proceeding to chest CT. Spirometry was performed using a portable spirometer by trained respiratory physiologists. Forced expiratory manoeuvres were performed according to ATS/ERS standards [15].

The decision to perform a chest CT scan was determined on an individual patient basis by a paediatric respiratory consultant, and based on a history of poor response to treatment and/or uncontrolled symptoms despite high levels of corticosteroid treatment. Every child who underwent chest CT scanning were on treatment step 4 or higher according to the 2014 British Thoracic Society asthma guidelines [16]. No child in our cohort had a prolonged wet cough. This case series study was registered as an audit with the University Hospitals of Leicester NHS Trust.

### Clinical characteristics

The electronic health records of all participants were reviewed to collect patient demographic data, information on prescribed asthma treatment, spirometry data, exhaled nitric oxide levels, total serum IgE, and induced sputum leukocyte differential cell counts. As this was a retrospective study, clinical data and chest CT scans were not acquired at the same time point. However we endeavoured to record clinical parameters which were acquired as closely as possible to the date of the CT scan.

### Controls

In order to compare differences in chest CT findings between asthma and non-asthmatic patients, we included the chest CTs of eight age matched controls who underwent CT imaging for cancer staging. None of the controls had a history of asthma or wheeze. None of the controls had evidence of thoracic malignancy on chest CT. Ethical approval was obtained from the Nottingham Research Ethics Committee 1 for control subjects (REC Ref: 09/H0403/92). Informed written consent was obtained from families before scoring CT scans.

### Chest computed tomography

Chest CT was performed using a Toshiba Aquilion 64 or GE Lightspeed 16 scanner. Sequential scanning was performed in patients with asthma at 10 mm increments with 1 mm collimation, from the apex of the lung to the diaphragm. As the age matched control subjects underwent CT scanning for the purpose of cancer staging, a volumetric spiral scan was performed. The number of CT slices obtained varied between patients based on their body habitus. Patients were scanned in the supine position at maximal inspiration, with their arms held over their head. Expiratory scans were obtained in asthma patients when specifically requested by the respiratory physician (22/30 images). Images were reconstructed using a high spatial frequency algorithm, through a 512 X 512 matrix, with a small field of view targeted to image only pulmonary areas. The peak voltage and effective tube current varied based on age and weight of the patient. Automatic tube current modulation was used to minimise the radiation dose. Images were saved and reported at a window width of 1600 Hounsfield units (HU) and a window level of -500HU [17].

### Image evaluation

The first step in our study protocol was to obtain all the control CT scans. Patients and parents were approached by their oncology consultant during routine follow-up clinic visits. After completion of control recruitment, the CT images of both DAC and control patients were pooled together and fully anonymised by a technician not involved with data collection or interpretation. Subsequently, all CT images were presented in a random order to two radiologists (SG and AM). Each radiologist independently evaluated and scored the CT images using a pre-agreed paediatric scoring system (Table 1) based on semi-quantitative assessment. The radiologists were blinded to the patient’s demographic data and clinical history. Only when all CT scans were uploaded were they analysed by the radiologists and scored; this is the reason why it was not possible to add scans after 2011, to ensure blinding of the radiologists scoring the CT scans of both asthma and control groups.

**Table 1:** Scoring System for CT Images

Severity of BE and BWT		Extent of BE and BWT		Extent of AT	
Score	Findings for BE or BWT	Score	Findings for BE or BWT	Score	Description of CT Findings for AT
0	None	0	No involvement	0	No increase in low attenuation on expiratory scan
1	Mild	1	< 1/3 lobe involvement	1	< 1/3 lobe involvement
2	Moderate	2	≥1/3 lobe involvement < 2/3 lobe involvement	2	≥1/3 lobe involvement < 2/3 lobe involvement
3	Severe	3	≥2/3 lobe involvement	3	≥2/3 lobe involvement

Scores were assigned individually for each individual lobe and for each lung separately (right upper, right middle, right lower, left upper, lingula and left lower). The scores from each lobe were added to obtain a composite score for each CT parameter. BE = bronchiectasis. BWT = bronchial wall thickening. AT = air trapping

When both inspiratory and expiratory phase scans were available, inspiratory phase scans were used for the assessment of BWT and BE. BWT (severity and extent), BE (severity and extent), extent of AT / mosaic attenuation and presence of mucoid impaction (MI) were assessed for each of the six lobes individually, including the lingula as a separate lobe. The scores from each lobe were then added to obtain a composite score for each CT parameter. As a result the composite score range was (i) 0–36 for BWT and BE extent and severity; (ii) 0–18 for AT / mosaic attenuation extent; and (iii) 0–6 for MI.

BE was considered to be present when the CT scan showed the presence of one or more of the following: [1] an internal diameter of the bronchus greater than that of the adjacent pulmonary artery; [2] a lack of tapering of the bronchial lumen toward the periphery; or [3] visualization of bronchus within 1 cm of the pleural surface [18]. Presence of AT was assessed in patients who underwent paired inspiratory and expiratory CT scans. Presence of mosaic attenuation was considered as a surrogate for AT when only inspiratory CT scans were available. Determination of the presence of BWT, AT / mosaic attenuation and MI were based on subjective assessment. The determination of severity of BE and BWT were also based on subjective assessment.

A score was assigned to severity and extent of BE and BWT in each lobe, and in extent only for AT / mosaic attenuation. Scores were independently assigned by the two radiologists. Disagreements were discussed and agreement reached by mutual consensus.

### Comparisons

Once all CT scores had been agreed and finalised, direct comparisons were made between the control and the DAC CT scores. Thereafter, in order to investigate associations between specific CT abnormalities and clinical and pathobiological parameters, the DAC patients were sub-divided into binary groups for each abnormality identified (i.e. BE versus no BE groups) so that comparisons could be made against recorded clinical variables including age, lung function, exhaled nitric oxide level, serum total IgE levels, and sputum leukocyte differential cell counts. Finally, the composite severity and extent scores assigned for each identified CT abnormality were correlated against clinical variables.

### Statistical analysis

All statistical analyses were carried out using IBM SPSS statistics for Windows software version 24.0 (Armonk, NY: IBM Corp), and GraphPad Prism version 7.00 for Windows (GraphPad Software, La Jolla California USA, www.graphpad.com). Descriptive statistics are expressed as the mean (SEM) or median (range) for continuous variables, and using percentages or proportions for categorical variables. Chi-squared test was used to compare categorical data. Continuous variables were compared using the independent T-Test for parametric data and the Mann-Whitney Test for non-parametric data. Correlations were performed using the Spearman rank correlation coefficient for non-parametric, and Pearson’s r correlation for parametric variables. Data was assessed for normality using the Shapiro-Wilk Test. Probability values of < 0.05 were considered statistically significant.

## Results

### Baseline characteristics

Thirty children (median age 12, range 5–16 years) were identified from our DAC register who underwent thoracic CT scans as part of their clinical workup for severe asthma between 2006 and 2011. A further eight age-matched children, without a history of asthma or wheeze, were recruited as controls. Control CT scans were performed between 2006 and 2014. Their characteristics are described in Table 2.

**Table 2:** Characteristics of DAC patients and non-asthmatic controls who underwent thoracic CT

Variable	DAC Patients (*n* = 30)	Controls (*n* = 8)	*P* value
Median Age in years (range)	12 (5–16)	12 (6–16)	0.994
Males	15/30 (50%)	6/8 (75%)	0.206
FEV_1_% predicted	82.1 (2.8)		
FVC % predicted	92.9 (2.7)		
FEV_1_/FVC %	73.6 (2.1)		
Exhaled Nitric Oxide (parts per billion)	66.8 (6.9)		
Sputum eosinophils (%)	16.9 (2.5)		
Sputum neutrophils (%)	46.7 (5.4)		
Total IgE	1206 (237)		

SEM is given unless otherwise indicated. *P*-value compares controls to DAC children

The asthma and control groups were well matched in terms of age and sex. No lung function data was available for the non-asthmatic controls as spirometry was not part of their routine clinical care.

### Main findings

Comparisons between the chest CT findings of the DAC patients and non-asthmatic controls are shown in Table 3.

**Table 3:** Prevalence of Abnormal CT Findings

	Controls (n = 8)	DAC Patients (n = 30)	P value
Bronchiectasis (BE) Present	0/8	8/30 (27%)	0.100
Bronchial Wall Thickening Observed (BWT)	1/8 (13%)	24/30 (80%)	< 0.001*
Mucoid Impaction Observed (MI)	1/8 (13%)	5/30 (17%)	0.774
Air Trapping Observed (AT)	0/8	18/30 (60%)	< 0.005*

*P < 0.05

**Fig. 1:**
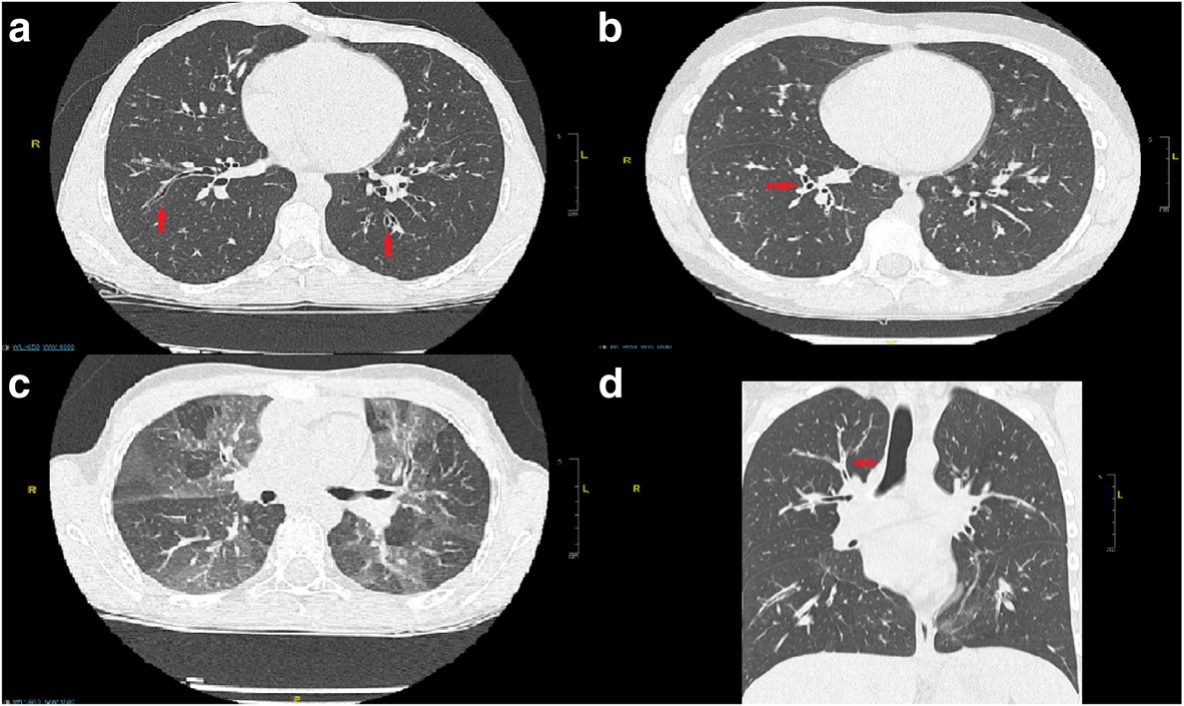
Examples of chest CT abnormalities found in our cohort of children with severe asthma: **a**) Bronchiectasis, **b**) Bronchial Wall Thickening, **c**) Air Trapping, and **d**) Mucous Plugging

In DAC patients, the most commonly observed abnormalities were BWT and AT. BWT was present in 80% of DAC patient scans and AT in 60%. BWT and AT both emerged as good discriminators between DAC patients and non-asthmatic controls. Mucoid impaction was observed in 17% of DAC patients and 13% of non-asthmatic control scans respectively, and was not a good discriminator between the two groups. BE was observed in eight out of the 30 (27%) DAC patients, but in none of the non-asthmatic controls. Examples of abnormal chest CT findings are shown in Fig. 1.

### Pattern of chest CT abnormalities in DAC patients

Of the 30 DAC patients, 26 (87%) had at least one radiological abnormality found on chest CT imaging. BWT co-existed with BE in 23% (7/30) of patients, BWT with AT in 57% (17/30) of patients, and AT with BE in 17% (5/30) of patients. BE, BWT and AT were observed together in 17% (5/30) of patients.

### Clinical determinants for CT abnormalities

Comparisons between DAC patients with and without BE, BWT, and AT are shown in Tables 4, 5 and 6. Leukocyte differential cell counts were missing from two DAC patients, and total IgE from one DAC patient.

**Table 4:** Comparison of DAC Patients with BE vs No BE

	BE (n = 8)	No BE (*n* = 22)	P-value
Serum IgE Level	1214 (356)	1203 (302)	0.984
Age	13.9 (0.67)	11.5 (0.61)	0.038*
Sputum Eosinophils (%)	22.8 (5.53)	14.8 (2.68)	0.162
Sputum Neutrophils (%)	56.1 (10.41)	43.9 (6.25)	0.349
FEV_1_% Predicted	87.4 (5.91)	80.2 (3.13)	0.267
FVC% Predicted	95.8 (3.92)	91.9 (3.46)	0.530
FEF_50_% Predicted	61.3 (6.42)	52.5 (4.33)	0.284
FEV_1_/FVC Ratio	0.80 (0.04)	0.71 (0.02)	0.068
Exhaled Nitric Oxide (ppb)	69.0 (14.19)	66.0 (8.14)	0.853

*P < 0.05 (Independent Sample T-Test). The data expressed as mean (SEM)

**Table 5:** Comparison of DAC Patients with BWT vs No BWT

	BWT (*n* = 24)	No BWT (*n* = 6)	P-value
Serum IgE Level	1354 (268)	494 (378)	0.175
Age	12.1 (0.52)	12.2 (1.62)	0.975
Sputum Eosinophils (%)	18.4 (2.82)	9.9 (4.33)	0.188
Sputum Neutrophils (%)	40.7 (5.90)	71.8 (3.44)	< 0.001*
FEV_1_% Predicted	82.7 (3.12)	79.8 (6.75)	0.688
FVC% Predicted	92.0 (3.25)	96.5 (4.26)	0.524
FEF_50_% Predicted	57.0 (3.93)	46.9 (8.86)	0.267
FEV_1_/FVC Ratio	0.75 (0.02)	0.66 (0.07)	0.107
Exhaled Nitric Oxide (ppb)	73.0 (7.56)	42.1 (13.9)	0.074

*P < 0.05 (Independent Sample T-Test). The data expressed as mean (SEM)

**Table 6:** Comparison of DAC Patients with AT vs No AT

	AT (*n* = 18)	No AT (*n* = 12)	P-value
Serum IgE Level	1152 (291)	1295 (422)	0.775
Age	11.5 (0.68)	13.1 (0.72)	0.132
Sputum Eosinophils (%)	20.5 (2.97)	11.5 (3.93)	0.075
Sputum Neutrophils (%)	37.0 (6.58)	60.0 (7.58)	0.032*
FEV_1_% Predicted	83.0 (3.80)	80.9 (4.20)	0.717
FVC% Predicted	92.6 (4.11)	93.4 (3.15)	0.892
FEF_50_% Predicted	56.9 (4.55)	52.1 (6.07)	0.521
FEV_1_/FVC Ratio	0.75 (0.02)	0.71 (0.05)	0.426
Exhaled Nitric Oxide (ppb)	72.1 (9.17)	58.8 (10.62)	0.358

*P < 0.05 (Independent Sample T-Test). The data expressed as mean (SEM)

**Fig. 2:**
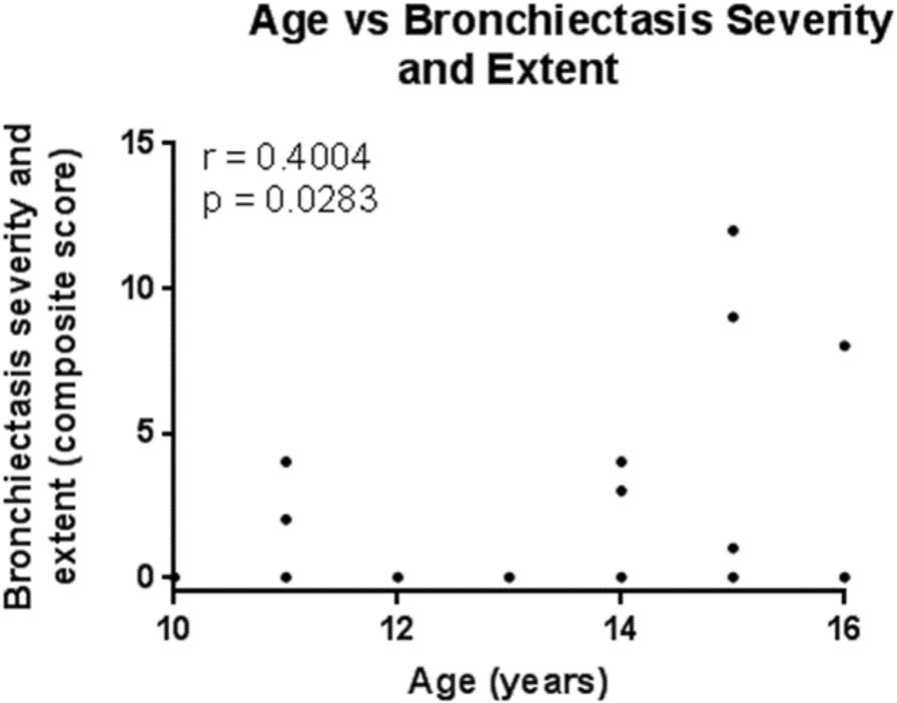
Scatter plot to demonstrate the relationship between age (years) and bronchiectasis severity and extent in children with severe asthma

DAC patients with evidence of BE on CT images were older than those without BE (13.9 ± 0.67 vs 11.5 ± 0.61, *p* = 0.038). There was also a positive correlation between increasing BE severity and extent with age (r = 0.400, *p* = 0.028) (Fig. 2). There was no difference in age between patients with positive vs negative findings of BWT or AT.

There was a trend towards raised total IgE, sputum eosinophil percentage on leukocyte differential cell counts, and exhaled nitric oxide levels being associated with BWT and AT; and raised total IgE levels with BWT.

Sputum eosinophil percentage cell count trended to be higher in the DAC patients with abnormal findings for BE (22.8 ± 5.53 vs 14.8 ± 2.68), BWT (18.4 ± 2.82 vs 9.9 ± 4.33) and AT (20.5 ± 2.97 vs 11.5 ± 3.93), but these did not reach statistical significance.

**Fig. 3:**
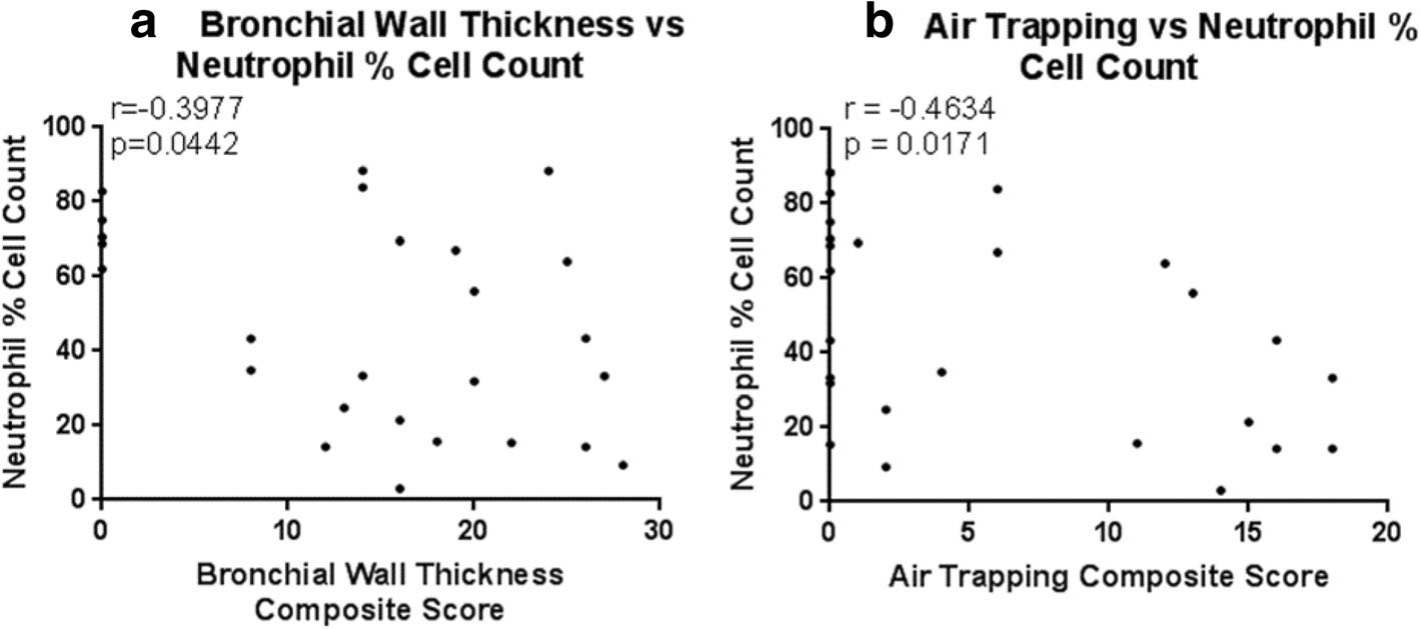
Fig. 3a+b. Scatter plots to demonstrate the relationship between neutrophil percentage cell count and bronchial wall thickness severity and extent, and between neutrophil percentage cell count and air trapping extent in children with severe asthma

Sputum neutrophil percentage cell count was lower in DAC patients with BWT and AT, and was negatively correlated to BWT severity and extent (r = − 0.398, *p* = 0.044), and AT extent (r = − 0.463, *p* = 0.017) (Fig. 3). In DAC patients with BE, mean neutrophil percentage cell count was higher compared to in patients without BE (56.1 ± 25.5 vs 43.9 ± 27.9), but this again did not reach statistical significance.

There were no differences in spirometry parameters observed between DAC patients with or without BE, BWT, or AT.

## Discussion

The role of chest CT imaging in the evaluation and management of children with severe asthma is unclear, and currently based on limited evidence. We conducted a retrospective study to quantify the prevalence of positive radiological findings in our cohort of children with severe asthma undergoing chest CT as part of their diagnostic work up, and to investigate their association with measured clinical parameters.

We found BE in approximately one third of children attending our difficult asthma clinic. This has not been reported in previous CT studies of children with severe asthma [11, 13]. A study by Marchac et al., which included 15 children with difficult-to-treat asthma receiving ≥800 mg/day beclomethasone dipropionate or budesonide for more than six months, did not identify CT evidence of MI, emphysema or BE [11] in any of their patients. The children in that study were of a similar age to our cohort but were on lower doses of inhaled corticosteroids and may therefore have had milder symptoms. In another study, Saglani et al. also did not find any evidence of BE on the chest CT images of 27 children with severe asthma that were only slightly younger than our cohort [13]. As not all of our paediatric patients with severe asthma routinely have chest CT scans, it may be that our selected cohort over-represented children with more severe disease. It is also worth noting that our study specifically sought to identify the presence of BE using a predefined criteria [18]. Moreover, the children with BE in our cohort were significantly older than those without BE. This is consistent with the higher prevalence of BE observed in studies of adult severe asthma patients, where the prevalence of BE is reportedly as high as 40% [9].

We found that abnormal chest CT findings were present in 87% of our paediatric severe asthma patients. BWT and AT were the most common abnormal findings, observed in 80 and 60% of our DAC patients respectively. No control CT scan showed evidence of AT, and BWT was only present in one non-asthmatic control scan. There was no difference in the prevalence of MI between our non-asthmatic controls and severe asthma patients. This may be because MI is a relatively common finding on chest CT (particularly with infections and malignancies), and is not specific to asthma [19]. Even though our control participants had no evidence of thoracic malignancy, it is likely that they would have been more vulnerable to infections as a result of their treatment, and therefore potentially more prone to chest infections.

We found no association between any spirometric parameter with either BE, BWT, or AT. Previous studies in children have yielded contradictory findings. One previous study in children reported that the degree of AT correlated with FEV_1_/FVC, RV, and TLC, but not with FEV_1_ [20]. Marchac et al. [11] and Saglani et al. [13]*,* by contrast, reported no correlation between HRCT scores for BWT or AT, with either FEV_1_ or FEF_25–75_. The authors concluded that respiratory function may not be sensitive to early airway structural changes. This view is also supported by a further paediatric study where Pifferi et al. [21] found that low density areas can be observed on chest CT images, similar to those observed in emphysema, in asthmatic children despite normal FEV_1_ and FEF_25–75_.

Adult studies have reported BE to be associated with poorer lung function [9], which was not observed in our cohort. It will be interesting to see if our DAC patients with BE have a faster rate of decline in lung function longitudinally when compared to those with no BE. Moreover, spirometry is not sensitive to early changes of lung damage [22], which may explain the lack of association observed between spirometric measures and the presence of BE within our cohort.

There was a trend towards raised markers of allergic disease and eosinophilic airway inflammation in DAC patients with BWT. Total IgE levels were three-times higher, and sputum eosinophil percentage cell count and exhaled nitric oxide levels approximately double in children with BWT compared to no BWT. Though this trend did not reach significance in our study, the authors of a previous paediatric study reported BWT was correlated with exhaled nitric oxide levels [12]. This is also in agreement with existing literature describing eosinophilic asthma as a distinct phenotype of asthma associated pathologically by thickening of the basement membrane zone [23].

The percentage of sputum neutrophils was found to be lower in our DAC patients with evidence of BWT or AT on CT. Even after correcting for multiple comparisons, the negative association with neutrophil percentage cell counts between BWT vs no BWT remained significant using an alpha level of 0.005 (*p* < 0.001). Moreover, we observed a significant, albeit weak, negative correlation between severity and extent of BWT, and extent of AT, with neutrophil percentage cell counts.

This suggests that high neutrophil percentage cell count was not associated with BWT in our patients. This is consistent with a previously described subgroup of adult patients with predominantly neutrophilic airway inflammation, absence of eosinophils and normal sub-reticular basement membrane thickness [24]. As discussed above, eosinophilic airway inflammation is associated with basement membrane thickening and BWT; and our data also demonstrated a trend of higher eosinophil percentage cell counts in children with BWT. Likewise, a previous study reported a significant negative correlation between sputum neutrophil and eosinophil percentage cell counts [25] in asthma patients. This may explain the negative association we observed between BWT and AT, with sputum neutrophil percentage cell count.

Another possible explanation is that sputum leukocyte profiles are unstable and have been shown to fluctuate over time; with one paediatric study reporting that sputum inflammatory phenotypes are not stable in 61% of children with asthma [26]. Hence the clinical significance of the apparent relationship between sputum neutrophil percentage cell count and CT findings in our study is unclear.

The main strength of our study was in having a control group, allowing blinding between non-asthmatic control and DAC patient scans during reporting. Only one previous study [11] included a control group. All chest CT scans were anonymised and reported independently by two radiologists. Disagreements were discussed and agreement reached by mutual consensus. Furthermore, well-recognized criteria were used for the identification of BE, which were decided upon a priori.

In terms of limitations, studies involving chest CT imaging of children with severe asthma are often limited by patient numbers, and ours was no exception. Also, whilst the presence of BE was definitely a feature in many of our children with severe asthma, the prevalence of BE may have been overestimated due to selection bias. Since patient selection for chest CT scanning was done at the discretion of the individual clinician managing the severe asthma patient, it is possible that only the most severely affected patients were imaged; therefore our findings may not be generalizable to all children with severe asthma.

Due to the retrospective nature of this study, the CT scanning parameters were not standardised between our healthy controls and asthma patients. We were also unable to ensure that all clinical measurements collected were performed at the same time as the chest CT scans. As measures of lung function and airway inflammation can be highly variable in children with asthma, this may explain why we found no correlation between CT findings and any spirometric parameter. Finally, the CT scans available for patients with severe asthma were sequential scans with 10 mm increments between each CT slice, thus limiting quantitative and semi-quantitative assessment of airway dimensions and AT. The CT scans were therefore assessed in a subjective manner which reflects current clinical practice.

Current guidelines recommend the use chest CT scans as a tool in the investigation of children with severe asthma, to exclude co-morbidities or to identify evidence of structural airway changes as these may impact the way the patient is managed. The high prevalence of abnormalities detected amongst our DAC patients would, at face value, support this recommendation. However, did these findings change how the patients were managed? Like previous studies in children with asthma [11, 13], we did not identify any alternative diagnoses to treat.

Although we did find a high proportion of DAC patients with BE, it is unclear whether BE observed in asthma patients represents a separate condition or natural progression of severe disease [27]. Either way there is currently no clear guidance on the management of BE in the context of severe asthma.

One case study, of an 11-year old boy with difficult asthma, reported significant improvement in asthma symptoms when BE was treated aggressively with physiotherapy, DNase, and antibiotics [28]. This raises the question of whether children with severe uncontrolled asthma and BE should be considered for appropriate treatment for non-CF BE. To our knowledge, this is not currently standard practice. In adults, there is evidence that regular macrolide add-on therapy is associated with reduced asthma exacerbations [29], at least in those with non-eosinophilic asthma [30]. This may be secondary to the anti-microbial or immunomodulatory effects of macrolides; and reflects their proven efficacy in other chronic airway diseases, including in patients with non-CF BE [31].

CT imaging in children is not risk free, and although the future cancer risk associated with low-level radiation (associated with CT) is not certain, expert panels agree that there is a small cancer risk which increases with increasing dose [32]. In view of the lack of alternative diagnoses identified, consideration needs to be given as to how CT chest imaging is likely to change management prior to requesting the investigation. Furthermore, if CT imaging in children with difficult asthma is to be justified, clear guidance of what to do when abnormal findings are detected needs to be in place.

Further research is needed to investigate the usefulness of chest CT imaging in children for 1) phenotyping asthma, 2) identifying patients at greater risk of lung function decline and, 3) guiding treatment algorithms stratified by CT abnormalities.

## Conclusion

In our study, we observed a high prevalence of bronchiectasis amongst a selected cohort of children with severe asthma. This has not been reported by previous paediatric studies. Abnormal CT findings are highly prevalent in nearly all our selected paediatric asthma population but these were not associated with lung function deficit, in agreement with previous studies. Importantly, we found no alternative diagnosis to asthma in any of our DAC children, also in accordance with previous studies. The decision of when to proceed to CT imaging remains unclear and needs to be made on the balance of radiation risk versus potential treatment benefits for the patient. A CT scan should only be proposed if it changes the management of the patient or in cases where an alternative diagnosis is suspected.

## Data Availability

The datasets used and analysed for this study are available from the corresponding author on reasonable request.

## Abbreviations

AT: Air Trapping
ATS: American Thoracic Society
BE: Bronchiectasis
BWT: Bronchial Wall Thickening
CT: Computed Tomography
DAC: Difficult Asthma Clinic
ERS: European Respiratory Society
FEV1: Forced Expiratory Volume in One Second
FVC: Forced Vital Capacity
HRCT: High Resolution Computed Tomography
HU: Hounsfield units
MI: Mucoid Impaction
SEM: Standard Error of the Mean
